# Melanoma Derived Exosomes Amplify Radiotherapy Induced Abscopal Effect via IRF7/I‐IFN Axis in Macrophages

**DOI:** 10.1002/advs.202304991

**Published:** 2024-01-29

**Authors:** Lu Wang, Kangjie Shen, Zixu Gao, Ming Ren, Chenlu Wei, Yang Yang, Yinlam Li, Yu Zhu, Simin Zhang, Yiteng Ding, Tianyi Zhang, Jianrui Li, Ming Zhu, Shaoluan Zheng, Yanwen Yang, Shisuo Du, Chuanyuan Wei, Jianying Gu

**Affiliations:** ^1^ Department of Plastic Surgery Zhongshan Hospital Fudan University Shanghai 200032 P. R. China; ^2^ Department of Plastic Surgery Shanghai Geriatric Medical Center Shanghai 201104 P. R. China; ^3^ Department of Plastic Surgery Zhongshan Hospital Xiamen Branch Fudan University Xiamen 361015 P. R. China; ^4^ Department of Radiotherapy Zhongshan Hospital Fudan University Shanghai 200032 P. R. China

**Keywords:** abscopal effect, I‐IFNs, IRF7, melanoma, radiotherapy

## Abstract

Radiotherapy (RT) can induce tumor regression outside the irradiation field, known as the abscopal effect. However, the detailed underlying mechanisms remain largely unknown. A tumor‐bearing mouse model is successfully constructed by inducing both subcutaneous tumors and lung metastases. Single‐cell RNA sequencing, immunofluorescence, and flow cytometry are performed to explore the regulation of tumor microenvironment (TME) by RT. A series of in vitro assays, including luciferase reporter, RNA Pulldown, and fluorescent in situ hybridization (FISH) assays, are performed to evaluate the detailed mechanism of the abscopal effect. In addition, in vivo assays are performed to investigate combination therapy strategies for enhancing the abscopal effect. The results showed that RT significantly inhibited localized tumor and lung metastasis progression and improved the TME. Mechanistically, RT promoted the release of tumor‐derived exosomes carrying circPIK3R3, which is taken up by macrophages. circPIK3R3 promoted Type I interferon (I‐IFN) secretion and M1 polarization via the miR‐872‐3p/IRF7 axis. Secreted I‐IFN activated the JAK/STAT signaling pathway in CD8^+^ T cells, and promoted IFN‐γ and GZMB secretion. Together, the study shows that tumor‐derived exosomes promote I‐IFN secretion via the circPIK3R3/miR‐872‐3p/IRF7 axis in macrophages and enhance the anti‐tumor immune response of CD8^+^ T cells.

## Graphical abstract

Radiotherapy (RT) has the potential to trigger tumor regression beyond the treated area, known as the abscopal effect. However, its underlying biological mechanism remains largely elusive. In this study, it is elucidated that RT can induce the abscopal effect in melanoma by utilizing tumor‐derived exosomes to activate the circPIK3R3/miR‐872‐3p/IRF7 pathway in macrophages, which stimulates the IFN secretion, enhancing the anti‐tumor immune response of CD8^+^ T cells.

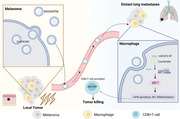

## 1 Introduction

Melanoma, a malignant cutaneous tumor, originates from melanocytes derived from the pluripotent cells of the neural crest. Melanoma is known for its high malignancy rate, accounting for 72% of skin tumor‐related deaths. Recently, its incidence has been increasing at a rate of 3–5% per year,^[^ ^1^ ^]^ making it one of the fastest‐growing malignancies. Although surgical treatment is effective for early‐stage melanoma, its aggressive nature and high propensity for early metastasis result in poor patient prognosis. The BRAF mutation rate in patients is approximately 41–55%.^[^ ^2^ ^]^ However, BRAF inhibitors, such as Dabrafenib and Trametinib (D+T), as well as immune checkpoint inhibitors (ICIs) targeting CTLA‐4 and PD‐1/PD‐L1, have not yielded optimistic outcomes in melanoma patients.^[^ ^3^, ^4^ ^]^ Therefore, the exploration of additional therapeutic approaches and the development of improved combination treatments are essential for improving patient prognosis.

Radiotherapy (RT) plays a crucial role in the treatment of patients with cancer, with more than half of patients with advanced tumors receiving RT as part of their therapeutic regimen. RT has been reported to produce highly satisfactory local control of melanoma.^[^ ^5^ ^]^ The mechanism of RT involves the induction of double‐stranded breaks (DSBs) in DNA, which leads to tumor cell necrosis and apoptosis.^[^ ^6^ ^]^ However, recent research has suggested that RT plays a crucial role in immune activation. Our previous study showed that RT promotes immune cloaking in hepatocellular carcinoma through the upregulation of PD‐L1 induced by cGAS‐STING and has the potential to enhance immunotherapy.^[^ ^7^ ^]^ Notably, in 1953, Mole et al. proposed the phenomenon of the RT‐induced abscopal effect, which refers to the regression of metastatic cancer at distant sites that are not directly irradiated.^[^ ^8^ ^]^ The abscopal effect is believed to be mediated by an immune response triggered by RT. When a local tumor is irradiated, it undergoes cellular stress or injury, leading to the release of neoantigens known as tumor‐associated antigens (TAAs). These TAAs are engulfed by antigen‐presenting cells (APCs) and subsequently presented to CD8^+^ T cells. CD8^+^ T cells recognize and attack both primary and metastatic tumors.^[^ ^9^ ^]^ However, the incidence of the abscopal effect remains low in clinical settings. Therefore, it is of great significance to explore the mechanism underlying the abscopal effect, increase its occurrence, and establish combined treatment strategies.

Macrophages are a major cell population in the tumor microenvironment (TME) and are enriched in RT‐treated tumor lesions.^[^ ^10^ ^]^ Recent studies have highlighted the involvement of macrophages in the abscopal effect. In breast cancer, HMGB1 and TNF‐α have been found to promote macrophage recruitment and M1 polarization, leading to a systemic anti‐tumor abscopal response triggered by RT.^[^ ^11^ ^]^ Additionally, hepatic CD169^+^ macrophages have been identified as a key factor responsible for the RT‐induced abscopal effect.^[^ ^12^ ^]^ Macrophages can be classified into two main phenotypes: M1 and M2. M1 polarization is associated with T‐cell activation and participates in anti‐tumor effects. Activation of M1 macrophages is mediated by cytokines such as IFN‐γ, GM‐CSF, TNF‐α, IL‐1, lipopolysaccharide (LPS), and extracellular matrix proteins.^[^ ^13^ ^]^ Recent studies reported that IRF7, a transcription factor crucial for the production of type I interferon (I‐IFN), contributes to M1 characteristics by activating the SAPK/JNK pathway.^[^ ^14^ ^]^ However, the detailed role of IRF7‐mediated M1 polarization in this process is currently not well understood.

In the present study, we investigated the potential role and mechanism of RT‐induced abscopal effect in melanoma. The specific biological mechanisms underlying the abscopal effect induced by RT were elucidated using single‐cell RNA sequencing (scRNA‐seq) and bulk RNA‐seq. Notably, this study revealed that after RT, melanoma cells released exosomes containing circPIK3R3, which promoted IRF7 expression and I‐IFN secretion via the miR‐872‐3p/IRF7 axis in macrophages, facilitating the abscopal effect. Importantly, we found that in individuals with low circPIK3R3 expression, the administration of RO8191 demonstrated the potential to enhance the abscopal effect of RT. Our findings provide valuable insights into the molecular mechanisms underlying the abscopal effect and a promising strategy for improving its efficacy.

## 2 Results

### 2.1 RT Inhibits Tumor Growth and Improves the TME of Local Melanoma

**Figure 1:**
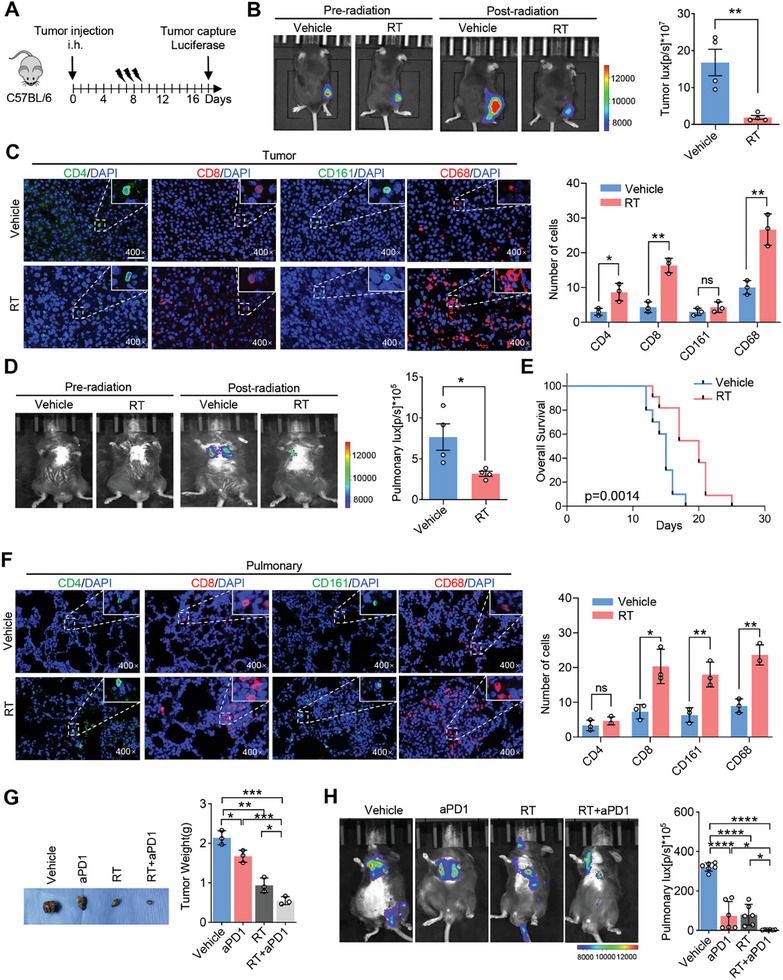
Effects of RT on the local tumor and distant lung metastases. A) Workflow of RT and bioluminescence imaging. B) Bioluminescence imaging showing the growth of subcutaneous melanoma tumors in the vehicle and RT groups, quantitatively analyzed based on bioluminescent signal intensity (*n* = 4) on day 7. C) Immunofluorescence analysis of CD4^+^ T cells, CD8^+^ T cells, NK cells (CD161C^+^ cells), and macrophages (CD68^+^ cells) infiltrating subcutaneous tumors in the vehicle and RT groups (*n* = 3) on day 7. D) Bioluminescence imaging depicting the growth of distant lung metastases in the vehicle and RT groups (*n* = 4) on day 18. E) Survival curves of mice in the vehicle and RT groups (*n* = 6). F) Immunofluorescence analysis of CD4^+^ T cells, CD8^+^ T cells, NK cells (CD161C^+^ cells), and macrophages (CD68^+^ cells) infiltrating distant lung metastases in the vehicle and RT groups (*n* = 3) on day 18. G) Measurement of subcutaneous tumor weight in each group to evaluate the therapeutic efficacy (*n* = 3). H) Evaluation of therapeutic efficacy by measuring the fluorescence intensity of lung metastases in mice using bioluminescence imaging (*n* = 6). Data are presented as mean ± SEM, ^*^ *p* < 0.05, ^**^ *p* < 0.01, ^***^ *p* < 0.001, ^****^ *p* < 0.0001 by two‐tailed unpaired Student t‐test.

To explore the effect of RT on melanoma, 1 × 106 B16F10‐luc cells were injected subcutaneously into C57/BL6 mice, which then received 8 Gy RT for three consecutive days from day 7 (**Figure** **1**A). Tumor volumes were measured using an in vivo imaging system before RT (day 6) and at the endpoint (day 18). We found that tumor volumes were significantly smaller in the RT group than in the vehicle group (Figure 1B). The effect of RT on the TME was investigated using immunofluorescence staining. We observed increased numbers of CD68^+^ macrophages, CD8^+^ T cells, and CD4^+^ T cells, whereas no differences in CD161^+^ NK cells were observed in the TME after RT (Figure 1C). Because CD8^+^ T cells play pivotal roles in exerting potent anti‐tumor effects, we further investigated their function using FCM. The results showed that the RT group exhibited an augmented presence of CD8^+^ T cells compared to the vehicle group in the TME (Figure S1A, Supporting Information). Notably, more TNF‐α^+^, Perforin^+^, PD1^+^, and Ki‐67^+^ CD8^+^ T cells were detected in the RT group compared to the vehicle group, indicating a stronger tumor‐killing ability (Figure S1B–E, Supporting Information). These results show that RT inhibited tumor growth and improved the TME of local melanoma.

### 2.2 RT Inhibits Tumor Growth and Improves the TME of Distant Lung Metastases

To investigate the impact of RT‐induced abscopal effects, we established an animal model with both primary tumors and distant lung metastases (Figure S2A, Supporting Information). Briefly, 1 × 106 B16F10 cells were subcutaneously inoculated into C57/BL6 mice. Next, 1 × 106 B16F10‐luc cells were intravenously administered via the tail vein on day 5. Lung metastases were assessed before RT by using an in vivo imaging system. Subsequently, mice in the radiotherapy group received RT on their subcutaneous tumors at a dose of 8 Gy on days 7–9. On day 18, lung metastases were assessed using an in vivo imaging system. The results showed that the tumor volumes were significantly lower in the RT group than in the vehicle group (Figure 1D). We also evaluated the effect of radiotherapy on the prognosis of these mice. Mice subjected to RT exhibited significantly prolonged survival times compared to those in the vehicle group (*p* = 0.0014) (Figure 1E).

Compared to the vehicle group, we observed a significant increase in the proportion of CD68^+^ macrophages, CD8^+^ T cells, and CD161^+^ NK cells in the TME of distant lung metastases, whereas no difference in CD4^+^ T cells was observed (Figure 1F). Using FCM, we demonstrated that the RT group exhibited an augmented presence of CD8^+^ T cells in the TME of distal lung metastases compared with the control group (Figure S2B, Supporting Information). Within these CD8^+^ T cells in distant lung metastases, a higher proportion of TNF‐α^+^, Perforin^+^, and Ki‐67^+^ cells were observed, indicating enhanced proliferation and cytotoxicity of CD8^+^ T cells in the RT group (Figure S2C–E, Supporting Information). However, there was no significant difference in the proportion of PD1^+^CD8^+^ T cells between the two groups (Figure S2F, Supporting Information). These results indicated that RT inhibited tumor growth and improved the TME of distant lung metastases.

### 2.3 Combination of RT and Anti‐PD1 Heightens the Abscopal Effect

Given that RT can improve the melanoma TME, we speculated that RT combined with anti‐PD1 may exert synergistic anti‐tumor effects. Here, we established a mouse model of the abscopal effect in which radiotherapy (8 Gy) was administered for three consecutive days from day 7, and anti‐PD1 therapy was administered once every two days starting from day 7 (Figure S3A, Supporting Information). We found that RT, anti‐PD1, and combination therapy inhibited local and distant tumor growth to different degrees; importantly, the combination therapy showed the most significant inhibitory effect on these tumors (Figure 1G,H). We detected CD8^+^ T cells in tumor tissues and found that combination therapy resulted in the highest CD8^+^ T cell infiltration in the TME (Figure S3B,C, Supporting Information). These results indicate that radiotherapy combined with anti‐PD1 improves the TME and heightens the abscopal effect in melanoma.

### 2.4 Irradiated Melanoma Cells Promote M1 Polarization via Upregulating IRF7 of Macrophages

**Figure 2:**
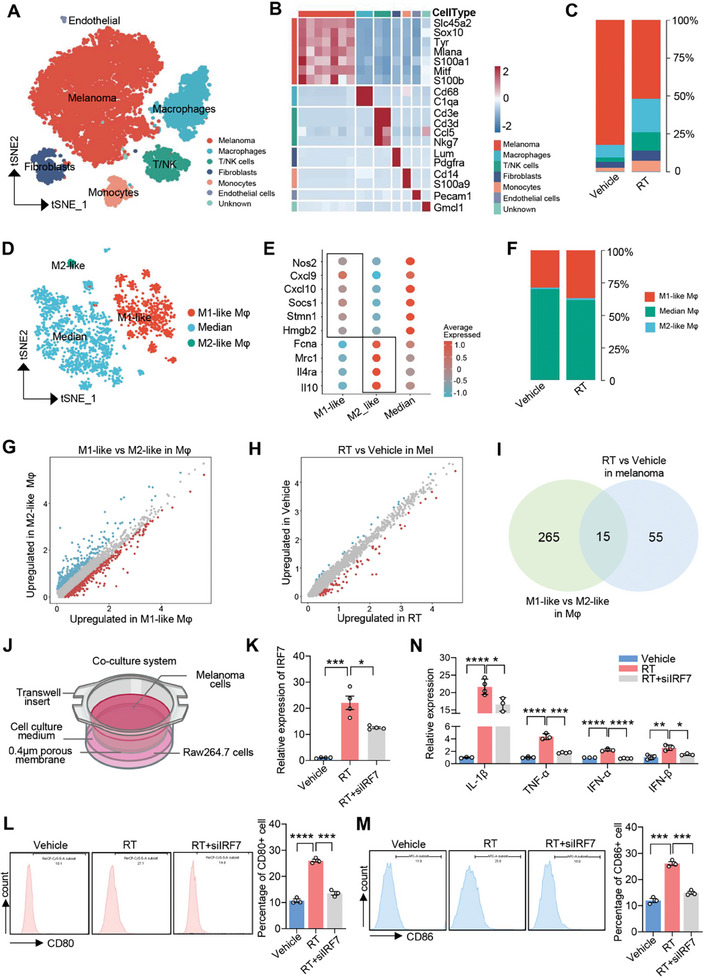
Irradiated melanoma cells promote M1 polarization and IRF7 expression of macrophages. A) t‐SNE plot illustrating the distribution of seven cell subpopulations. B) Heatmap depicting the expression levels of marker genes for each cell subpopulation. C) Percentage of different cell types in the vehicle and RT groups (*n* = 3). D) t‐SNE plot displaying the distribution of three macrophage subpopulations. E) Expression profile of marker genes in the three macrophage subpopulations. F) Percentage of the three macrophage subpopulations in the vehicle and RT groups. G) Volcano plot depicting differentially expressed genes between M1‐like macrophages and M2‐like macrophages, with log2FC > 0.45 and p‐value < 0.05. H) Volcano plot illustrating differentially expressed genes of melanoma cells between the vehicle and RT groups, with log2FC > 0.45 and p‐value < 0.05. I) Venn diagram displaying the overlapping differentially expressed genes upregulated in both RT‐treated melanoma cells and M1‐like macrophages. J) Schematic representation of the co‐culture model of macrophages and RT‐treated melanoma cells. K) qRT‐PCR analysis of IRF7 expression in macrophages. L,M) FCM analysis of CD80 and CD86 expression in macrophages. N) qRT‐PCR analysis of IL‐1β, TNF‐α, IFN‐α, and IFN‐β expression in macrophages. Data in (J–M) are presented as mean ± SEM, *n* = 3. ^*^ *p* < 0.05, ^**^ *p* < 0.01, ^***^ *p* < 0.001, ^****^ *p* < 0.0001 by two‐tailed unpaired Student t‐test.

To understand the mechanism of action of RT, we performed scRNA‐seq on melanoma tissues with and without RT. After rigorous data quality control and preprocessing, 12517 cells meeting the inclusion criteria were selected for subsequent analyses. Based on the classical marker genes,^[^ ^15^, ^16^ ^]^ these cells were categorized into seven distinct cell types: melanoma cells (Slc45a2, Sox10, Tyr, Mlana, S100a1, Mitf, and S100b), macrophages (Cd68 and C1qa), T/NK cells (Cd3e, Cd3d, Ccl5, and Nkg7), fibroblasts (Lum and Pdgfra), monocytes (Cd14 and S100a9), endothelial cells (Pecam1), and other cells (**Figure** **2**A,B). The proportion of melanoma cells decreased, whereas the percentages of macrophages, T/NK cells, monocytes, and fibroblasts increased in the RT group (Figure 2C). Macrophages, which play important roles in tumor progression, were the most abundant immune cell types in the melanoma TME (Figure 2C). We further characterized the macrophages based on their marker gene expression,^[^ ^17^ ^]^ and identified three distinct types: M1‐like macrophages (high expression of Nos2, Cxcl9, Cxcl10, Socs1, Stmn1, and Hmgb2), M2‐like macrophages (high expression of Fcna, Mrc1, Il4ra, and Il10), and intermediate‐type macrophages (Figure 2D,E). Intriguingly, our results revealed that the number of M1‐like macrophages significantly increased in the RT group (Figure 2F).

To describe the functional characteristics of macrophages, we conducted a differential expression analysis between M1‐like and M2‐like macrophages and found that 280 genes were upregulated in M1‐like macrophages (log2FC > 0.45, p < 0.05) (Figure 2G). Gene Ontology (GO) and Kyoto Encyclopedia of Genes and Genomes (KEGG) analyses unveiled several enriched functions in M1‐like macrophages, including the IFN‐α response, lymphocyte migration, myeloid differentiation, B‐cell differentiation, lymphocyte apoptosis, and fatty acid oxidation (Figure S2A, Supporting Information). To further explore the detailed mechanism of M1‐like polarization, we performed a correlation analysis of gene expression profiles among different cell types and found a positive correlation in gene expression between macrophages and melanoma cells/T/NK cells (Figure S2B, Supporting Information). Here, we speculated that irradiated melanoma cells may regulate the biological functions of macrophages. We conducted a differential expression analysis to compare the gene expression profiles of melanoma cells with and without RT. Remarkably, we identified 70 genes that were significantly upregulated in melanoma cells following radiotherapy (log2FC > 0.45, *p* < 0.05) (Figure 2H). To explore the potential communication between melanoma cells and macrophages after RT, we performed an intersection between these upregulated genes in RT‐treated melanoma cells and M1‐like macrophages. Fifteen genes were identified, including Fcer1g, Psmb8, Tmsb4x, Bst2, Psme1, Psmb9, Ifit3, Isg15, Mlana, Psme2, Psmb10, Irf7, Cxcl10, Gbp2, and Ccl5 (Figure 2I). Among them, IRF7 stands out as a crucial transcription factor in type I interferons (I‐IFNs) production, which was consistent with the results of GO analysis in Figure S2A (Supporting Information). Notably, it has been established that IRF7 can upregulate the co‐stimulatory molecule CD80 and promote the secretion of pro‐inflammatory cytokines such as IL‐12 and IL‐15 in macrophages, thereby driving their polarization toward the M1 phenotype.^[^ ^18^ ^]^ We hypothesized that irradiated melanoma cells induce an M1‐like phenotype in macrophages by upregulating the expression of IRF7.

To investigate the crosstalk between the irradiated melanoma cells and macrophages, we established an in vitro co‐culture system (Figure 2J). We observed elevated IRF7 expression in macrophages co‐cultured with irradiated melanoma cells (Figure 2K). Accordingly, elevated CD80 and CD86 levels were observed in macrophages co‐cultured with irradiated melanoma cells (Figure 2L,M). Meanwhile, increased pro‐inflammatory factors, including IL‐1β, TNF‐α, IFN‐α, and IFN‐β, were also presented in macrophages co‐cultured with irradiated melanoma cells (Figure 2N). However, IRF7 knockdown with small interfering RNA (siRNA) significantly inhibited these inflammatory factors (IL‐1β, TNF‐α, IFN‐α, and IFN‐β), CD80 and CD86 in macrophages co‐cultured with irradiated melanoma cells (Figure 2L–N). These results indicate that irradiated melanoma cells promote the M1‐like polarization of macrophages by upregulating IRF7 expression.

### 2.5 Irradiated Melanoma‐derived Exosomes Induce Macrophage M1 Polarization via Upregulating IRF7

**Figure 3:**
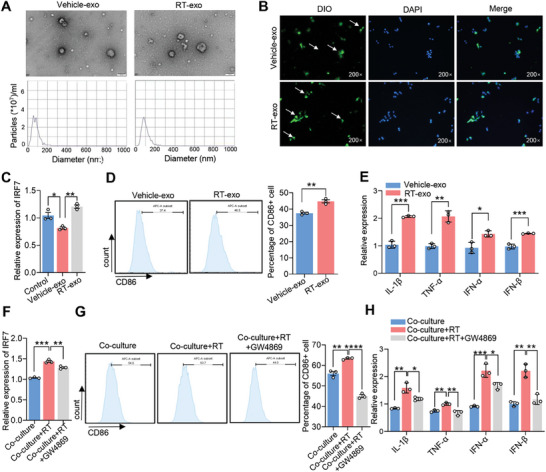
Irradiated melanoma‐derived exosomes induce macrophage M1 polarization via upregulating IRF7. A) Electron microscopy and NTA analysis of exosomes released by vehicle and RT‐treated melanoma cells. B) Uptake of DiO‐labeled exosomes released by melanoma cells by macrophages. C) qRT‐PCR analysis of IRF7 expression in macrophages induced by exosomes released from RT‐treated melanoma cells. D) FCM analysis of CD86 expression on the cell surface of macrophages induced by exosomes released from RT‐treated melanoma cells. E) qRT‐PCR analysis of IL‐1β, TNF‐α, IFN‐α, and IFN‐β expression in macrophages induced by exosomes released from RT‐treated melanoma cells. F) qRT‐PCR analysis of IRF7 expression in macrophages after treatment with GW4869. G) FCM analysis of CD86 expression on the cell surface of macrophages after treatment with GW4869. H) qRT‐PCR analysis of IL‐1β, TNF‐α, IFN‐α, and IFN‐β expression in macrophages after treatment with GW4869. Data in (C–H) are presented as mean ± SEM, *n* = 3. ^*^ *p* < 0.05, ^**^ *p* < 0.01, ^***^ *p* < 0.001, ^****^ *p* < 0.0001 by two‐tailed unpaired Student t‐test.

Numerous studies have shown that tumor‐derived exosomes can reprogram the TME.^[^ ^19^ ^]^ Here, we further speculate that irradiated melanoma cells may promote macrophage M1 polarization by secreting exosomes. To test this hypothesis, we isolated exosomes secreted by melanoma cells with or without radiotherapy. We performed electron microscopy to detect the characteristics of the exosomes and found that these exosomes exhibited a cup‐ and saucer‐shaped structure with a double‐layer membrane‐like configuration, ranging in diameter from to 40–160 nm. There were no differences in exosome morphology and size between the RT and vehicle groups (**Figure** **3**A, upper). Subsequently, nanoparticle tracking analysis (NTA) confirmed that the particle sizes of the exosomes in both groups were predominantly concentrated within the range of 30–200 nm (Figure 3A, lower). To determine whether these melanoma‐derived exosomes (melanoma‐Exos) could be taken up by macrophages, we stained the extracted exosomes from both groups with DIO and co‐cultured them with macrophages. We found that DIO‐labeled melanoma‐Exos from both the vehicle and radiotherapy groups were present in the cytoplasm of macrophages, indicating that melanoma‐Exos could be absorbed by macrophages (Figure 3B). These findings strongly suggest that macrophages are capable of efficiently removing the exosomes released by melanoma cells.

Then we treated macrophages with B16F10‐Exos in the RT and vehicle groups at a concentration of 10 µg mL^−1^. Macrophages were collected for FCM and qRT‐PCR analyses. IRF7 levels were significantly upregulated in macrophages treated with B16F10‐Exos in the RT group (Figure 3C). Remarkably, macrophages in the RT group exhibited an upregulation of CD86 expression, as well as increased levels of IL‐1β, TNF‐α, IFN‐α, and IFN‐β (Figure 3D,E). These results suggest that B16F10‐Exos in the RT group were responsible for IRF7 upregulation in macrophages, leading to M1 polarization. To clarify the role of exosomes in M1 polarization, we used GW4869, a sphingomyelinase inhibitor of exosome secretion. GW4869 (10 µm) was added into the co‐culture system of melanoma cells and macrophages and incubated for 24 h. Interestingly, IRF7 expression in macrophages was significantly downregulated by GW4869 treatment (Figure 3F). The levels of CD86, IL‐1β, TNF‐α, IFN‐α, and IFN‐β were also downregulated in macrophages after GW4869 treatment (Figure 3G,H). These results indicate that irradiated melanoma cells promote IRF7 expression in macrophages by releasing exosomes.

Surprisingly, there was no difference in IRF7 mRNA expression between exosomes released by irradiated and non‐irradiated melanoma cells (Figure S5A, Supporting Information). This suggests that there may be other components in exosomes, rather than IRF7 mRNA itself, that mediate IRF7 mRNA upregulation in macrophages.

### 2.6 Exosomal circPIK3R3 Promotes M1 Polarization via Upregulating IRF7

**Figure 4:**
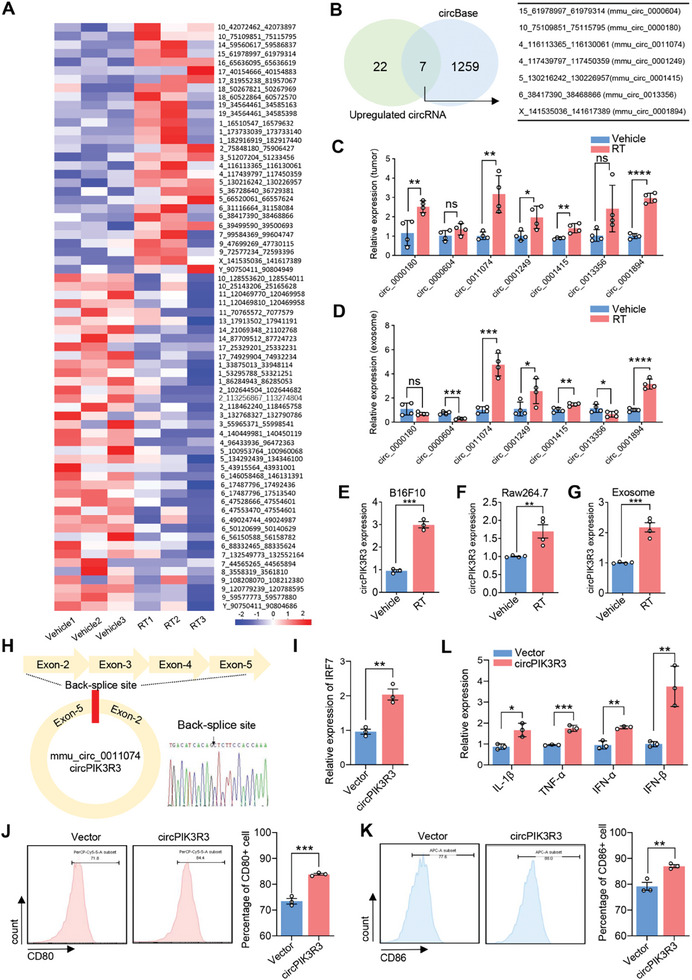
Exosomal circPIK3R3 promotes M1 polarization via upregulating IRF7 in macrophages. A) Heatmap displaying differentially expressed circRNAs in subcutaneous tumors of vehicle and RT groups, with logFC > 1.5 and p_adjust < 0.05 (*n* = 3). B) Comparison with the circBase circular RNA database revealed that out of the 1266 circRNAs detected by whole transcriptome sequencing in subcutaneous tumors of mice, 29 differentially expressed circRNAs in the RT group had corresponding identifiers and sequences in the circBase database. Venn diagram shows that seven up‐regulated circRNAs in the subcutaneous tumors of the RT group had matching identifiers and sequences in the circBase database. C) qRT‐PCR analysis of differentially expressed circRNAs in subcutaneous tumors of vehicle and RT groups (*n* = 4). D) qRT‐PCR analysis of differentially expressed circRNAs in peripheral blood exosomes (*n* = 4). E) qRT‐PCR analysis of circPIK3R3 expression in RT‐treated melanoma cells. F) Expression of circPIK3R3 in macrophages co‐cultured with RT‐treated melanoma cells. G) Expression of circPIK3R3 in exosomes derived from RT‐treated melanoma cells. H) Sanger sequencing revealing the back‐splicing site of circ_0011074. I) qRT‐PCR analysis of IRF7 expression in macrophages overexpressing circPIK3R3. J,K) FCM analysis of CD80 and CD86 expression in macrophages overexpressing circPIK3R3. L) qRT‐PCR analysis of IL‐1β, TNF‐α, IFN‐α, and IFN‐β expression in macrophages overexpressing circPIK3R3. Data are presented as mean ± SEM, *n* = 3. ^*^ *p* < 0.05, ^**^ *p* < 0.01, ^***^ *p* < 0.001, ^****^ *p* < 0.0001 by Student's t‐test.

Exosomes contain various substances, including circular RNA (circRNA), miRNA, proteins, and other components.^[^ ^20^ ^]^ In the present study, we investigated melanoma‐derived exosomes that promote IRF7 mRNA expression and M1 polarization of macrophages after RT. However, there was no significant difference in the IRF7 mRNA levels in the exosomes between the vehicle and RT groups. CircRNAs are strongly resistant to exonucleolytic degradation and maintain high cellular stability because of their covalently closed‐loop structure. Recently, numerous studies have shown that exosomal circRNAs play important roles in tumor progression and TME reprogramming. Here, we further deduced that melanoma‐derived exosomal circRNAs, rather than the mRNA itself, affect macrophage polarization. To explore potential circRNAs, we conducted whole‐transcriptome sequencing of tumor tissues obtained from the vehicle and RT groups. We identified 68 differentially expressed circRNAs (logFC > 1.5, *p* < 0.05), of which 29 were upregulated and 39 were downregulated (**Figure** **4**A). Among these, only seven upregulated circRNAs in the RT group were queried in the circBase database (Figure 4B). Next, we examined the upregulated circRNAs in the tumor tissues and peripheral blood exosomes. QRT‐PCR showed that circ_0011074, circ_0001249, circ_0001415, and circ_0001894 were upregulated in tumor tissues (Figure 4C) and peripheral blood exosomes (Figure 4D). Among them, circ_0011074 showed the highest expression and was selected for further study. We detected the expression of circ_0011074 in the co‐culture system and confirmed that it was upregulated in B16F10 cells, Raw264.7, and B16F10‐Exos in the RT group compared to that in the vehicle group (Figure 4E–G). These results indicated that circ_0011074 may participate in the regulation of M1 polarization in the co‐culture system.

Sanger sequencing confirmed that circ_0011074 originated from the cyclization of exons 2–5 of PIK3R3 (Figure 4H). We established a stable transfected Raw264.7 cell line with circPIK3R3 overexpression and the efficiency was determined by qRT‐PCR (Figure S6A, Supporting Information). Using qRT‐PCR analysis, we demonstrated that circPIK3R3 overexpression promoted IRF7 mRNA expression in macrophages (Figure 4I). Meanwhile, elevated circPIK3R3 promoted the M1 polarization of macrophages, as detected by increased expression CD80 and CD86 (Figure 4J,K), and IL‐1β, TNF‐α, IFN‐α, and IFN‐β (Figure 4L). These findings strongly suggest that exosomal circPIK3R3 promotes M1 polarization by upregulating IRF7 expression in macrophages.

### 2.7 CircPIK3R3 Upregulates IRF7 Expression via Sponging miR‐872‐3p

**Figure 5:**
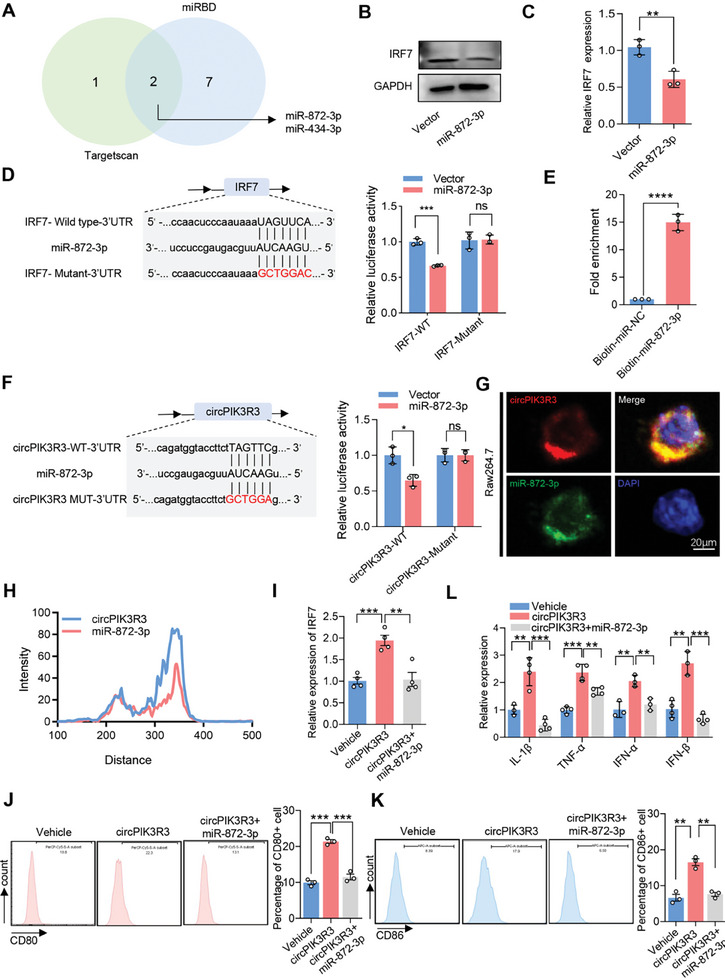
CircPIK3R3 upregulates IRF7 expression via sponging miR‐872‐3p. A) Venn diagram displaying the miRNAs that target IRF7 according to TargetScan and miRBD databases. B,C) Western blot and qRT‐PCR analysis examining the effect of miR‐872‐3p overexpression on IRF7 expression. D) Binding sites of miR‐872‐3p with IRF7. Dual‐luciferase reporter gene assay was performed to assess the binding relationship between miR‐872‐3p and IRF7. E) RNA Pull‐down assay detecting the expression level of CircPIK3R3 bound to biotinylated miR‐872‐3p probe. F) Binding sites of CircPIK3R3 with miR‐872‐3p. Dual‐luciferase reporter gene assay was conducted to evaluate the binding relationship between CircPIK3R3 and miR‐872‐3p. G) FISH analysis revealing the spatial relationship between CircPIK3R3 and miR‐872‐3p in Raw264.7 cells. H) Fluorescence signal quantifification according to the location of circPIK3R3 and miR‐872‐3p in (G). I) qRT‐PCR analysis of IRF7 expression in Raw264.7 cells treated with Vector, circPIK3R3, and circPIK3R3+miR‐872‐3p. J,K) FCM analysis of CD80 and CD86 expression in Raw264.7 cells treated with Vector, circPIK3R3, and circPIK3R3+miR‐872‐3p. L) qRT‐PCR analysis of IL‐1β, TNF‐α, IFN‐α, and IFN‐β expression in Raw264.7 cells treated with Vector, circPIK3R3, and circPIK3R3+miR‐872‐3p. Data are presented as mean ± SEM, *n* = 3. ^*^ *p* < 0.05, ^**^ *p* < 0.01, ^***^ *p* < 0.001, ^****^ *p* < 0.0001 by Student's t‐test (C–F) and two‐tailed unpaired Student t‐test (I–K).

Several studies have shown that circRNAs promote tumor progression through miRNA sponges.^[^ ^21^ ^]^ Here, we speculate that circPIK3R3 upregulates IRF7 expression by sponging certain miRNAs. We identified potential miRNAs that could bind to the 3′ end of IRF7 using targetscan and miRBD databases, and found that miR‐872‐3p and miR‐434‐3p may be potential target (**Figure** **5**A). We found that IRF7 mRNA and protein levels were significantly decreased in cells transfected with miR‐872‐3p mimics (Figure 5B,C), whereas no significant decrease was observed in cells transfected with miR‐434‐3p mimics (data not shown). Accordingly, we identified a putative binding site between miR‐872‐3p and IRF7 mRNA, which was selected for further validation (Figure 5D). We performed a luciferase reporter assay using cells transfected with a vector containing either wild‐type IRF7 (IRF7‐WT) or a mutated miR‐872‐3p binding site (IRF7‐Mut). Following transfection with miR‐872‐3p mimics, the luciferase reporter activity was significantly decreased in IRF7‐WT cells, while no significant reduction was observed in IRF7‐Mut cells, indicating that miR‐872‐3p binds to the predicted site in the 3′ end of IRF7 (Figure 5D). These findings indicate that miR‐872‐3p inhibits IRF7 expression by directly binding to its target site.

To explore the potential interaction between miR‐872‐3p and circPIK3R3, we designed a specific biotinylated miR‐872‐3p probe to perform RNA Pulldown assay and found that the biotinylated miR‐872‐3p probe could effectively capture circPIK3R3 (Figure 5E). We performed luciferase reporter assays by transfecting cells with luciferase reporter vectors (containing the wild‐type or mutant sequence of the miR‐872‐3p target). Compared with the mutant sequence, luciferase reporter activity was significantly decreased by miR‐872‐3p mimics in cells transfected with the wild‐type sequence (Figure 5F). Additionally, fluorescent in situ hybridization (FISH) showed that circPIK3R3 and miR‐872‐3p were mainly located in the cytoplasm of Raw264.7 cells, and importantly, they were clearly co‐localized. (Figure 5G–H). These findings indicate that miR‐872‐3p directly binds to circPIK3R3 at the target site.

To determine whether circPIK3R3 regulated IRF7 expression via the miR‐872‐3p sponge, we performed rescue assays. In Raw264.7 cells, overexpression of circPIK3R3 promoted IRF7 expression, which was reversed by miR‐872‐3p mimics (Figure 5I). Accordingly, high levels of M1 markers CD80 and CD86, and pro‐inflammatory factors IL‐1β, TNF‐α, IFN‐α, and IFN‐β, were also presented in circPIK3R3 overexpressed macrophages, and these were reversed by miR‐872‐3p mimics (Figure 5J–L). Taken together, these results confirm that circPIK3R3 promotes the M1 polarization of macrophages via the miR‐872‐3p/IRF7 axis.

### 2.8 CircPIK3R3 Promotes Macrophage I‐IFN Secretion to Activate CD8^+^ T Cells

**Figure 6:**
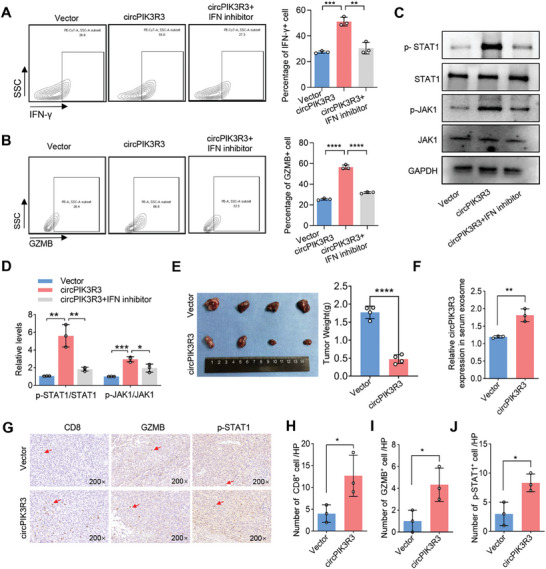
CircPIK3R3 promotes macrophage I‐IFN secretion to induce CD8^+^ T cell activation. A,B) FCM analysis of IFN‐γ and GZMB expression in CD8^+^ T cells co‐cultured with macrophages. C,D) Western blot analysis showing the activation of the JAK1‐STAT1 pathway in CD8^+^ T cells co‐cultured with macrophages. E) Subcutaneous tumor model assessing the impact of circPIK3R3 on melanoma growth by measuring tumor weight (*n* = 4). (F) qRT‐PCR detection of CircPIK3R3 expression in peripheral blood exosomes of mice (*n* = 3). G–J) Immunohistochemical examination of CD8^+^ T cell infiltration, GZMB expression, and p‐STAT1 expression in melanoma tissues. Data are presented as mean ± SEM, *n* = 3. ^*^ *p* < 0.05, ^**^ *p* < 0.01, ^***^ *p* < 0.001, ^****^ *p* < 0.0001 by two‐tailed unpaired Student t‐test (A–D) and Student's t‐test (E–J).

Considering that IRF7 can promote I‐IFN secretion and M1 polarization of macrophages, all of which can activate T cells, we further speculated that macrophage overexpressed circPIK3R3 could promote CD8^+^ T cell activation. After co‐culturing CD8^+^ T cells with circPIK3R3 overexpressed macrophages, we detected higher levels of IFN‐γ and GZMB in CD8^+^ T cells. This was largely reversed by the IFN inhibitor, indicating circPIK3R3 induces CD8^+^ T cell activation mainly via promoting macrophage I‐IFN secretion (**Figure** **6**A,B). Higher levels of phosphorylated STAT1 (p‐STAT1) and JAK1 (p‐JAK1), which are key signaling molecules downstream of the I‐IFN pathway, were present in the circPIK3R3 overexpression group, and were also restrained by the IFN inhibitor (Figure 6C,D).

We then generated a stably transfected B16F10 cell line overexpressing circPIK3R3 and verified its transfection efficiency using qRT‐PCR (Figure S7A, Supporting Information). B16F10‐circPIK3R3 and B16F10‐Vector cells were inoculated subcutaneously into C57/BL6 mice. Remarkably, the tumor weight in the B16F10‐circPIK3R3 group was lower than that in the B16F10‐Vector group (Figure 6E). There was a notable increase in circPIK3R3 levels in the peripheral blood exosomes of the B16F10‐circPIK3R3 group (Figure 6F). Using immunohistochemical assay, we showed that increased CD8^+^ T cells and enhanced GZMB and p‐STAT1 levels were present in the B16F10‐circPIK3R3 group compared to in the B16F10‐Vector group (Figure 6G–J). Collectively, these findings indicate that the overexpression of circPIK3R3 effectively inhibits the progression of melanoma and promotes the activation and infiltration of CD8^+^ T cells.

### 2.9 RT Exerts a Synergistic Anti‐Tumor Effect with Anti‐PD1

**Figure 7:**
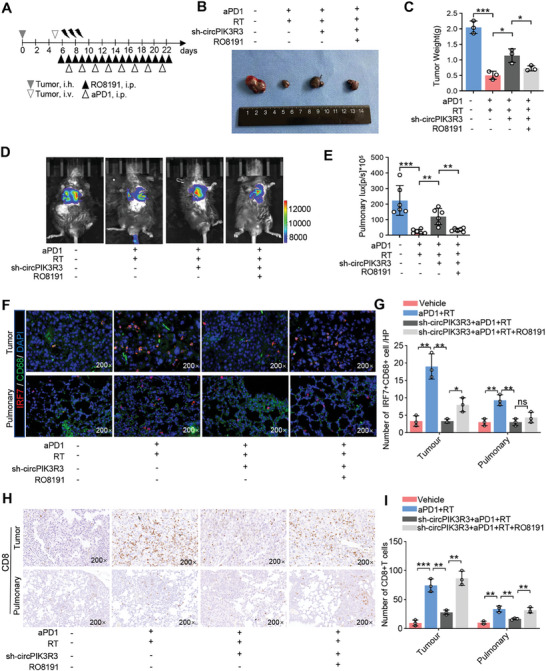
CircPIK3R3/IRF7/I‐IFN axis participates in the combination of radiotherapy and anti‐PD1 mediated abscopal effect. A) The treatment model involving RT, anti‐PD1, and RO8191: C57/BL6 mice were subcutaneously inoculated with 1 × 106 sh‐NC B16F10 cells or sh‐circ‐0011074 B16F10 cells. On day 5, C57/BL6 mice were intravenously injected with 1 × 106 B16F10‐luc cells. Starting from day 6, mice were administered the IFN receptor agonist RO8191 via daily intraperitoneal injections at a dose of 1 mg kg^−1^. On day 7, radiotherapy was initiated, with a daily dose of 8 Gy administered for 3 consecutive days. On day 7, mice were also administered anti‐PD1 via intraperitoneal injection every 2 days at a dose of 100 µg/mouse until the endpoint of observation. B,C) Measurement of subcutaneous tumor weight in each group to assess treatment efficacy (*n* = 3). D,E) Evaluation of fluorescent intensity in lung metastatic foci using bioluminescence imaging to assess treatment efficacy (*n* = 6). F,G) Immunohistochemical examination of CD8^+^ T cell infiltration in subcutaneous tumors and lung metastatic foci (*n* = 3). H,I) Immunofluorescence detection of IRF7^+^ macrophage infiltration in subcutaneous tumors and lung metastatic foci (n = 3). Data are presented as mean ± SEM. ^*^ *p* < 0.05, ^**^ *p* < 0.01, ^***^ *p* < 0.001, ^****^ *p* < 0.0001 by two‐tailed unpaired Student t‐test.

To investigate the role of circPIK3R3 in the combination of RT and anti‐PD1, we generated a B16F10 cell line with a circPIK3R3 knockdown and verified its knockdown efficiency using qRT‐PCR (Figure S7B, Supporting Information). Then C57/BL6 mice were divided into four groups: the Vehicle group, RT+anti‐PD1 group, shcircPIK3R3+RT+anti‐PD1 group, and shcircPIK3R3+RT+anti‐PD1+RO8191 (I‐IFNR agonist) group; the treatment procedure is depicted in the schematic diagram (**Figure** **7**A). We observed that circPIK3R3 knockdown significantly inhibited the combination therapy (RT and anti‐PD1)‐induced abscopal effect, whereas this effect was reversed by RO8191, an I‐IFN agonist capable of reactivating the I‐IFN pathway triggered by circPIK3R3 (Figure 7B–E). Meanwhile, we detected IRF7^+^ macrophages in the TME and found that circPIK3R3 knockdown significantly inhibited their infiltration after combination therapy (RT and anti‐PD1), and this was also reversed by RO8191 (Figure 7F,G). This finding is consistent with a previous study that showed a strong positive feedback loop between IRF7 and I‐IFN.^[^ ^22^ ^]^ Additionally, the combination of anti‐PD1 and RT significantly increased the number of CD8^+^ T cells in the TME, whereas circPIK3R3 knockdown compromised the effect of RT and anti‐PD1 treatment on CD8^+^ T cell infiltration in both local tumors and distant lung metastases. Furthermore, supplementation with the I‐IFNR agonist restored CD8^+^ T‐cell infiltration (Figure 7H,I). These results indicate that the circPIK3R3/IRF7/I‐IFN axis participates in the abscopal effect mediated by the combination of RT and anti‐PD1 agents.

## 3 Discussion

**Figure 8:**
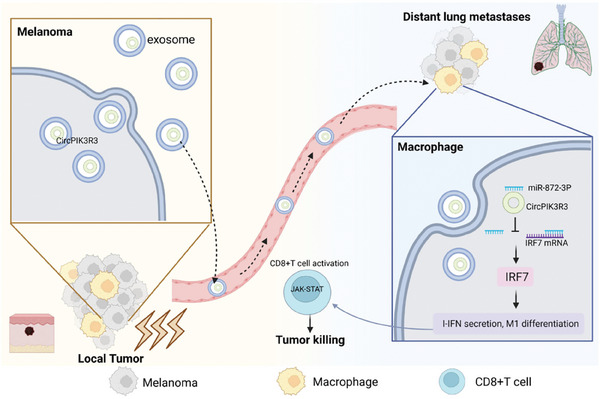
A schematic diagram depicting the biological function and mechanism of circPIK3R3 in RT‐induced abscopal effect in melanoma.

The abscopal effect, a phenomenon observed during RT, has significant implications for the prognosis of patients with advanced tumors. However, the mechanism underlying the abscopal effect remains unclear. In this study, we constructed a mouse model of RT‐induced abscopal effects and elucidated the biological function of RT in facilitating exosome secretion from melanoma cells. Exosomes carrying circPIK3R3 trigger M1 polarization and promote I‐IFN secretion via the miR‐872‐3p/IRF7 axis in macrophages. Additionally, secreted I‐IFN activated the JAK1‐STAT1 signaling pathway in CD8^+^ T cells, leading to increased secretion of IFN‐γ and GZMB. The circPIK3R3/IRF7/I‐IFN axis plays a crucial role in regulating RT‐induced abscopal effects by facilitating the infiltration of CD8^+^ T cells and IRF7^+^ macrophages into local tumors and distant lung metastases (**Figure** **8**). Furthermore, in individuals with low circPIK3R3 expression, administration of RO8191 demonstrated the potential to enhance the abscopal effect. These findings provide valuable insights into the molecular mechanisms underlying the abscopal effect and suggest a promising strategy for improving the efficacy of RT.

Currently, animal models of abscopal effects mainly use subcutaneous transplant tumors on both sides of the back.^[^ ^23^ ^]^ However, in clinical practice, melanoma mainly metastasizes to the lungs, liver, brain, and other organs, which significantly affects patient prognosis. Therefore, it is important to investigate whether radiotherapy has an abscopal effect on these vital organs. Schrand et al. developed an animal model by irradiating subcutaneous tumors and measuring lung weight to observe the impact on non‐irradiated lung metastases.^[^ ^24^ ^]^ This study found that the lung tissue weight in the radiation group was lower than that in the control group but higher than that in the normal lung tissue. However, lung weight does not accurately reflect the progression of lung tumors. In the present study, we used an animal model that simulates primary lesions by subcutaneous inoculation of B16F10 cells and used intravenous injection of B16F10‐luc cells to induce lung metastasis. The growth of lung metastases was evaluated using an in vivo imaging system. This model directly reflects the progression of lung metastases and eliminates the interference from subcutaneous tumor metastasis. Our findings demonstrated that RT effectively suppressed the growth of both local melanoma and distant lung metastases and prolonged the survival time of these tumor‐bearing mice.

Studies have shown that RT can reprogram the TME and enhance the anti‐tumor immune response. For example, Monjazeb et al. reported increased numbers of CD8^+^ T cells and PD1^+^Ki67^+^CD8^+^ T cells in the radiation field after hypofractionated radiation therapy (HFRT).^[^ ^25^ ^]^ In another study, Jarosz‐Biej et al. observed that after treatment with a single dose of 10 Gy RT, CD8^+^ T cells and macrophages increased, while M2‐like macrophages and angiogenesis decreased.^[^ ^26^ ^]^ In our study, we observed increased infiltration of CD8^+^ T cells, CD4^+^ T cells, and macrophages in local tumors following RT. Moreover, macrophages were polarized toward the M1‐like phenotype. Our findings are generally consistent with those from previous studies, although there are some differences in the details, which may be attributed to the different tumor types. These consistent results support the reliability of our conclusions.

Macrophages represent the predominant cell type within the TME and play a crucial role in TME reprogramming and tumor progression. In earlier studies, our research team discovered that after RT, tumor cells undergo necrosis, leading to the release of dsDNA and inducing the production of IFN‐β and TNF‐α in macrophages via the cGAS/STING axis, resulting in the positive feedback between iRhom2 and TNF‐α.^[^ ^27^ ^]^ RT‐induced macrophage polarization is regulated by factors such as radiation dosage and fractionation scheme. For example, low‐dose irradiation programs macrophage differentiation into an iNOS^+^/M1 phenotype that orchestrates effective T‐cell immunotherapy.^[^ ^28^ ^]^ In oral squamous cell carcinoma, local irradiation causes vascular damage and hypoxia in the tumor and increases the infiltration of CD11b^+^ myeloid cells, which are characteristic of M2 macrophages and associated with the promotion of vascularization.^[^ ^29^ ^]^ Our research revealed that after RT, there is strong crosstalk between tumor cells and macrophages, leading to macrophage polarization. These changes are characterized by the upregulation of CD80 and CD86 and secretion of inflammatory cytokines, indicative of M1 polarization, all of which result in improvements in TME and tumor regression.

Tumor cells can regulate the TME via multiple means, such as by releasing exosomes. For example, pancreatic cancer exosomes initiate premetastatic niche formation in the liver. Pancreatic ductal adenocarcinoma (PDAC)‐ derived exosomes highly express macrophage migration inhibitory factor (MIF), promoting liver metastasis and potentially serving as a prognostic marker for PDAC liver metastasis.^[^ ^30^ ^]^ In another study, Chao et al. demonstrated that RT‐MPs (RT‐microparticles) polarize microenvironmental M2 tumor‐associated macrophages (M2‐TAMs) to M1‐TAMs and modulate anti‐tumor interactions between TAMs and tumor cells.^[^ ^31^ ^]^ We found that melanoma‐derived exosomes induced M1 polarization of macrophages via IRF7 upregulation after RT. Extracellular vesicles (EVs) contain various substances, such as proteins, lipids, and RNA. Among them, circRNA is generated through the back‐splicing of precursor mRNA. It is stable, resistant to degradation, and involved in diverse biological regulation within the TME, including immune surveillance, angiogenesis, extracellular matrix remodeling, and resistance to radiation and chemotherapy.^[^ ^32^ ^]^ Our previous study on melanoma showed that circ_0020710 acts as a sponge for miR‐370‐3p, leading to increased CXCL12 expression.^[^ ^33^ ^]^ This in turn reduces the infiltration of CD8^+^ T cells and induces the formation of an immunosuppressive microenvironment. In the present study, we conducted whole‐transcriptome sequencing and validated our findings both in vitro and in vivo that circPIK3R3 promotes M1 polarization by upregulating IRF7 in macrophages. There are several mechanisms through which circRNAs regulate gene transcription. For instance, circRNAs can recruit functional proteins,^[^ ^34^ ^]^ facilitating their binding to the promoter region of target genes, or circRNA can directly bind to mRNA, thereby modulating the transcription of target genes.^[^ ^35^ ^]^ However, the mechanism that has been extensively investigated is the “sponge” function of circRNA, when circRNA acts as a miRNA sponge to sequester miRNAs and regulate the transcription of target genes. We performed luciferase reporter, FISH, RNA pulldown, western blotting, and PCR assays, and validated that circPIK3R3 upregulates IRF7 levels by sponging miR‐872‐3p.

Recent studies have reported an association between circRNAs and radiation response. Liu et al. demonstrated that circRNA_100367 regulates the radiation sensitivity of esophageal squamous cell carcinomas through the miR‐217/Wnt3 pathway.^[^ ^36^ ^]^ Another study reported that the circRNA TUBD1, which is significantly upregulated in irradiated LX‐2 Cells, acts as an miR‐146a‐5p sponge to influence the viability and pro‐inflammatory cytokine production of LX‐2 Cells through the TLR4 Pathway.^[^ ^37^ ^]^ However, there are no reports on the role of circRNAs in radiation‐induced abscopal effects. Our findings indicate that knockdown of circ_0011074 inhibits the effects of combined radiotherapy and immunotherapy on both local tumors and distant lung metastases, suggesting its involvement in regulating radiation‐induced abscopal effects. This highlights its potential as a biomarker for predicting the efficacy of the abscopal effects. An increasing number of studies have discovered that the combination of RT and immunotherapy significantly increases the occurrence rate of abscopal effects, for example, the combination of α‐OX40 co‐stimulation and radiation is a viable approach to overcome therapeutic resistance to PD‐1 blockade in immunologically cold tumors, such as triple‐negative breast cancer.^[^ ^38^ ^]^ The combination of cisplatin, RT, and PD‐1 blockade enhances the occurrence of abscopal effects. This effect is achieved by promoting CD8^+^ T cell activation via the CXCR3/CXCL10 axis.^[^ ^39^ ^]^ In our study, we found that in individuals with low circPIK3R3 expression, treatment with RO8191 (an I‐IFN agonist) enhanced the therapeutic effects of radiation‐induced abscopal effects. It increases the infiltration of CD8^+^ T cells into both subcutaneous tumors and lung metastases. These findings may lead to a novel combination therapeutic strategy for patients with advanced melanomas.

However, this study has several limitations. First, it is important to investigate whether there are substances in EVs other than circRNA, that contribute to the regulation of the TME and generation of abscopal effects. Second, radiation‐induced abscopal effects should be clinically validated in patients with melanoma. Further research is required to address these issues.

In conclusion, our study elucidates the role of exosomal circPIK3R3 in regulating the macrophage IRF7/I‐IFN axis, thereby enhancing the abscopal effects of radiotherapy in melanoma. Furthermore, we demonstrated that the administration of RO8191 improved the abscopal effect of RT in individuals with low circPIK3R3 expression. These findings provide valuable insights into the mechanisms underlying the abscopal effect and offer potential strategies for enhancing its clinical efficacy.

## 4 Experimental Section

### Cell Lines and Transfection

B16F10, Raw264.7, and 293T cells were purchased from the cell bank of the Chinese Academy of Sciences (Shanghai, China) and cultured under recommended conditions. All the lentiviral vectors used in this study were purchased from Obio Technology (Shanghai, China). The circPIK3R3‐overexpression and circPIK3R3‐shRNA lentiviral vectors were transfected into B16F10 cells and Raw264.7 cells, and blank lentiviral vectors were used as negative controls. The circPIK3R3 shRNA sequences used in this study are listed in Table S3 (Supporting Information). Transfection efficiency was evaluated using qRT‐PCR. A small interfering RNA targeting IRF7 with the sequence CCAGAUGCGUGUUCCUGUA was designed and synthesized by Genomeditech (Shanghai, China). miR‐872‐3p mimics and control plasmids were purchased from Genomeditech (Shanghai, China) and transiently transfected with Lipofectamine 2000 reagent (Invitrogen, USA) according to the manufacturer's instructions.

### Mouse Model and Treatment

Male 5‐6‐week‐old C57BL/6 mice were purchased from the Beijing Viton Lever Company. The mice were housed under specific pathogen‐free conditions at the Experimental Animal Center of Zhongshan Hospital, Fudan University. Experiments were conducted in accordance with the guidelines approved by the Experimental Animal Protection, Welfare, and Ethics Committee of Zhongshan Hospital, Fudan University. B16F10 cells (1 × 106) and their derivatives (B16F10‐luc, B16F10‐vector, B16F10‐circPIK3R3, or B16F10‐shcircPIK3R3) were injected subcutaneously into the right flank of C57/BL6 mice. B16F10‐luc cells (1 × 106) were intravenously administered via the tail vein on day 5. On day 7, when the tumors reached an average size of 60–80 mm^3^, the animals were randomly assigned to the control and different treatment groups. All mice (including mice receiving sham radiation) were anesthetized by intraperitoneal (i.p.) injection of pentobarbital sodium (20 mg kg^−1^), and the primary tumors were irradiated with three fractions of 8 Gy on days 7, 8, and 9 using the Small Animal Radiation Research Platform (300 cGy min^−1^, 6‐MeV‐ray beam; Oncor, Siemens). Anti‐mouse PD‐1 mAb (Bio X Cell, USA) was administered intraperitoneally (200 mg per mouse) every 2 days during treatment. RO8191 (Cayman Chemical, Ann Arbor, MI, USA) was administered intraperitoneally (100 mg/mouse) daily from day 6. Bioluminescence imaging was performed using an IVIS Lumina K Series III imaging system, and the image radiance values were normalized using Living Image software (PerkinElmer, Boston, MA, USA).

### Immunohistochemical (IHC) Assay

The tissue specimens were sliced and sequentially immersed in xylene and various concentrations of ethanol for deparaffinization. Subsequently, the slices were placed in an antigen retrieval buffer containing EDTA (pH 8.0). Bovine serum albumin solution (BSA) was added for blocking and incubated for 60 min. The slices were then incubated overnight at 4 °C with a diluted primary antibody (listed in Table S2, Supporting Information). Next, a diluted horseradish peroxidase‐conjugated secondary antibody was added, and the slices were incubated at room temperature for 1 h. Sections were then stained with diaminobenzidine, counterstained with hematoxylin, dehydrated in ethanol, cleared in xylene, and cover‐slipped with resin. Finally, the slides were analyzed using the ImageJ software.

### Immunofluorescence (IF) Assay

Tissues were embedded and sectioned with 0.1% Triton X‐100 permeabilization for 10 min, and blocked with 5% bovine serum albumin (BSA)at 37 °C for 60 min. Then sections were incubated with primary antibody (listed in Table S2, Supporting Information) overnight at 4 °C, then incubated with secondary antibody conjugated with Alexa Fluor‐488 or −594 for 1 h at room temperature. Finally, sections were stained with 4′, 6‐diamidino‐2‐phenylindole (DAPI, Yeasen, Shanghai, China) for 5 min and evaluated using laser confocal microscopy (Leica TCS SP5 II, Wetzlar, Germany). Mean values were calculated from three randomly selected microscopic fields from each section of each animal. Three animals were analyzed per group. Data are expressed as the mean number of positive cells per high‐power field.

### Flow Cytometry (FCM)

Tumor tissue was minced, digested with collagenase IV (Sigma), and incubated with RBC lysis buffer (BD Biosciences) to lyse the red blood cells. The resulting single‐cell suspension was washed and resuspended in phosphate‐buffered saline (PBS) containing 0.1% BSA. Subsequently, membrane markers were stained with fluorochrome‐conjugated antibodies and incubated at 4 °C for 40 min. The single‐cell suspension was also stained for intracellular proteins using corresponding fluorochrome‐conjugated antibodies and the Fixation/Permeabilization Solution Kit (BD Biosciences). FACS data were collected using a BD FACS Celesta instrument and analyzed using FlowJo V.10.0. Detailed information regarding the FCM antibodies is provided in Table S2 (Supporting Information).

### Single Cell RNA Sequencing (scRNA‐Seq)

Tumor tissues were dissociated into single‐cell suspensions, and the quality‐checked suspensions were quantified for cell viability, ensuring a minimum of 85%, using the 10x Genomics Chromium system. Single cells were then encapsulated into Gel Bead in Emulsion (GEMs) and subjected to reverse transcription using a PCR instrument. Following the oil‐breaking treatment, the amplified cDNA was purified using magnetic beads, and the subsequent steps included cDNA amplification and quality assessment. Libraries were constructed according to the manufacturer's protocol. The final library pool was sequenced on an Illumina Nova6000 instrument using 150‐base‐pair paired‐end reads.

### ScRNA‐Seq Data Preprocessing

Raw data were analyzed using the Cell Ranger software. Quality control was conducted using the Seurat package, when genes expressed in fewer than two cells were filtered out, and cells meeting the criteria of >200 genes and ≤20% mitochondrial genes were selected for subsequent analysis. Seurat package functions, including normalizeData, FindVariableFeatures, and ScaleData, were used to calculate the coefficient of variation for the genes. Principal Component Analysis (PCA) was performed on the expression profiles of the top 2000 highly variable genes, and the top 50 principal components were selected for subsequent dimensionality reduction clustering. Batch effects between the samples were corrected using the Harmony package. The clustering results were then visualized using t‐SNE (t‐distributed Stochastic Neighbor Embedding). Individual cells were manually annotated based on classical marker genes of each cell subpopulation. Functional analysis of cellular marker genes involved Gene Ontology (GO) and Kyoto Encyclopedia of Genes and Genomes (KEGG) enrichment analyses. Marker genes for each cluster (min.pct = 0.25, logfc.threshold = 0.25) were identified using the FindAllMarkers function in the Seurat package. KEGG enrichment analysis provided insights into the functional pathways enriched in each cell subpopulation.

### Preparation of Purified CD8^+^ T Cells

CD8^+^ T cells (Miltenyi, Germany) were obtained from the spleens of mice by negative selection using magnetic cell separation, according to the manufacturer's instructions. Then, CD8^+^ T cells were cultured for one week in a medium containing IL‐2 (10 ng mL^−1^), anti‐CD3 (4 µg mL^−1^), and anti‐CD28 (1 µg mL^−1^), and prepared for further study.

### Coculture Assay

For the co‐culture system of macrophages and melanoma cells, Raw264.7 cells were inoculated into the upper chamber of 0.4 µm Transwell, and B16F10 cells were inoculated into the lower chamber of 0.4 µm Transwell for different treatments. Subsequent experiments were performed after 48 h. For the co‐culture system of CD8^+^ T cells and macrophages, CD8^+^ T cells were inoculated into the upper chamber of a 0.4 µm Transwell and inoculated with macrophages; subsequent experiments were performed after 48 h.

### Exosome Extraction

Exosomes from mouse serum and melanoma cell culture medium were isolated using ExoQuick Exosome Precipitation Solution (Umibio, Shanghai, China) according to the manufacturer's instructions.

### Exosome Identification

For nanoparticle tracking analysis (NTA) assay, samples were diluted with PBS and analyzed using a NanoSight NS300 instrument (NanoSight, Malvern, PA, United Kingdom). For transmission electron microscopy (TEM) method, samples were fixed with 2% paraformaldehyde for 2 h. Subsequently, they were loaded onto a thin‐film copper‐mesh electron microscope grid, stained with 2% uranyl acetate for 2 min, and imaged under an electron microscope operating at 1200 kV.

### Exosome Uptake Assay

Raw264.7 cells were seeded into six‐well plates and incubated. Exosomes pretreated with DiO (5 µm) were added to culture medium and incubated at 37 °C for 8 h. The cells were washed with PBS, fixed in 4% formaldehyde, stained with DAPI, and visualized using fluorescence microscopy.

### Luciferase Reporter Assay

Luciferase reporter vectors were synthesized by Genomeditech (China). 293T cells were seeded into 96‐well plates and co‐transfected with a luciferase reporter vector and miR‐872‐3p mimics or a negative control using the Lipofectamine 2000 transfection reagent (Invitrogen, USA). After 48 h, firefly and Renilla luciferase activities were measured using a Dual‐Luciferase Kit (Promega, France) according to the manufacturer's instructions.

### Fluorescent In Situ Hybridization (FISH)

For FISH assay, circPIK3R3 (circPIK3R3 probe sequence: 5′‐ TTGGTGGAAGAGCTGTGATGTCCTTGT‐3′) and miR‐872‐3p (miR‐872‐3p probe sequence 5′‐AGGAGGCTACTGCAATAGTTCA‐3′) were captured with Alexa Cy3‐labeled and 488‐labeled probes (Obio Technology, Shanghai, China) respectively. After pre‐hybridization, circPIK3R3 and miR‐872‐3p probes were hybridized in the prepared hybridization buffer, and the nuclei were stained with DAPI (Yeasen, Shanghai, China), a confocal microscope was used to obtain images.

### RNA Pull‐Down

Biotin‐labeled miR‐872‐3p and negative control (NC) were transfected into Raw264.7 cells. Following transfection, the cells were lysed using a Lysis Buffer. The lysate was mixed with RNA‐RNA Hybridization Buffer (1:2 ratio) and a biotin‐labeled probe (Biotin‐miR‐872‐3p‐Probe sequence: ugaacuauugcaguagccuccu). The solution was thoroughly mixed and incubated overnight at room temperature. Subsequently, streptavidin magnetic beads were incubated with the mixture for 4 h. The beads were then washed, and TRIZOL was added to extract RNA. QRT‐PCR was performed to detect the expression level of circPIK3R3.

### Immunoblotting Assay

For western blotting, total protein extracts from the cells were separated by sodium dodecyl sulfate‐polyacrylamide gel electrophoresis (SDS‐PAGE), transferred onto polyvinylidene difluoride membranes. After blocking with 5% BSA, the membranes were incubated with primary antibody overnight at 4 °C. The membranes were then incubated with secondary antibody for 60 min. The bands were incubated with an ECL kit and analyzed using an imaging system. Densitometric analysis was performed using the ImageJ software. Detailed information on the immunoblotting assay is presented in Table S2 (Supporting Information).

### QRT‐PCR

Total RNA was extracted using TRIzol reagent (Invitrogen, USA). The extracted RNA was then reverse‐transcribed to cDNA using a PrimeScript RT Reagent Kit (EZ Bioscience, China) according to the manufacturer's protocol. qRT‐PCR was conducted using a SYBR Green Master MIX Kit (EZ Bioscience, China) according to the manufacturer's instructions. The qRT‐PCR assays were performed in triplicate, and the relative gene expression was determined using the 2^−ΔΔCt^ method. The primers used are listed in Table S1 (Supporting Information).

### Statistical Analysis

Statistical analyses were performed using the SPSS software (version 19.0; SPSS, Inc., Chicago, IL, USA) or GraphPad Prism 7.0 (GraphPad Software, USA). Data are presented as mean ± standard deviation. In this study, various statistical methods were employed for data analysis, including the unpaired Student's t‐test, Mann–Whitney U‐test, one‐way ANOVA test, Kruskal–Wallis rank‐sum test, and Spearman correlation analysis. The cumulative survival time was estimated using the Kaplan–Meier estimator, with significance assessed through the log‐rank test. Unless explicitly specified, a two‐sided test was utilized. Results were considered statistically significant if the *P*‐value was <0.05.

## Conflict of Interest

The authors declare no conflict of interest.

## Author Contributions

L.W., K.S., Z.G., and M.R. contributed equally to this work. C.W. and S.D. designed the study, L.W., M.R., Z.G., and C.W. completed the experimental process, K.S., Y.L., J.L., and Y.Y. conducted the statistical analyses, L.W., Y.Z., Y.D., and T.Z. wrote the paper, J.G., M.Z., S.Z., S.M.Z., and Y.Y. revised the paper. All authors reviewed the manuscript. All authors read and approved the final manuscript.

## Supporting information

Supporting Information

## Data Availability

The data that support the findings of this study are available from the corresponding author upon reasonable request.
