# Supplementary material for: Melanoma Derived Exosomes Amplify Radiotherapy Induced Abscopal Effect via IRF7/I‐IFN Axis in Macrophages
Source: Adv Sci (Weinh). 2024 Jan 29;11(13):2304991. doi: 10.1002/advs.202304991 (PMC10987102; doi:10.1002/advs.202304991)
Supplement: Supplementary file 1 — Supporting Information [file ADVS-11-2304991-s001.pdf]

ADVANCED SCIENCE
Open Access

Supporting Information

for *Adv. Sci.*, DOI 10.1002/advs.202304991

Melanoma Derived Exosomes Amplify Radiotherapy Induced Abscopal Effect via IRF7/I-IFN Axis in Macrophages

Lu Wang, Kangjie Shen, Zixu Gao, Ming Ren, Chenlu Wei, Yang Yang, Yinlam Li, Yu Zhu, Simin Zhang, Yiteng Ding, Tianyi Zhang, Jianrui Li, Ming Zhu, Shaoluan Zheng, Yanwen Yang, Shisuo Du, Chuanyuan Wei* and Jianying Gu**

Figure S1

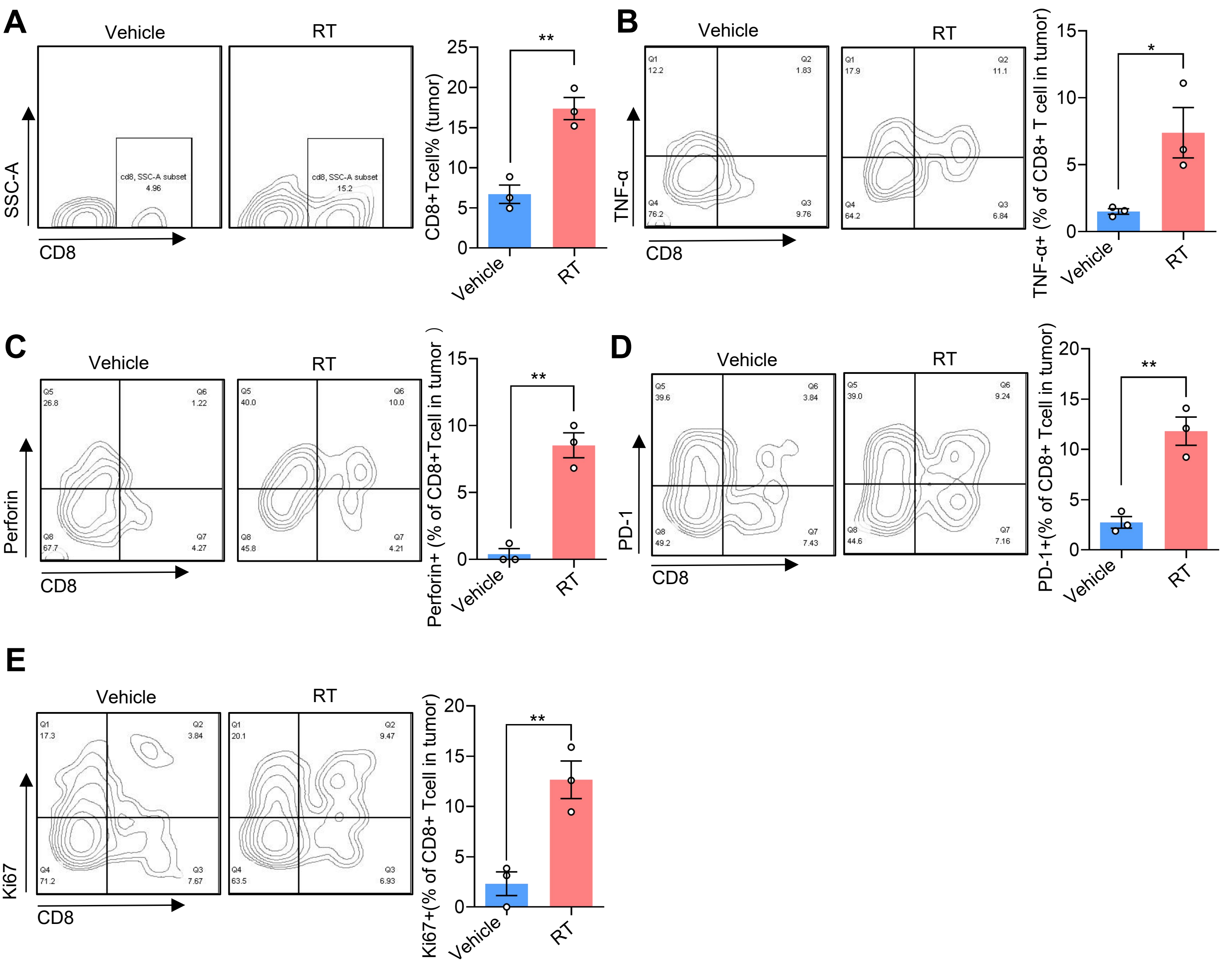

Figure S1 Effect of RT on CD8⁺ T cell infiltration in local tumors. (A) FCM analysis of CD8⁺ T cell infiltration in melanoma tissues in the vehicle and RT groups. (B-E) FCM analysis of TNF-α, Perforin, PD-1, and Ki-67 expression in CD8⁺ T cells in subcutaneous tumors of the vehicle and RT groups. Data are presented as mean ± SEM, n = 3. *$p < 0.05$, **$p < 0.01$, ***$p < 0.001$, ****$p<0.0001$ by Student's t-test.

Figure S2

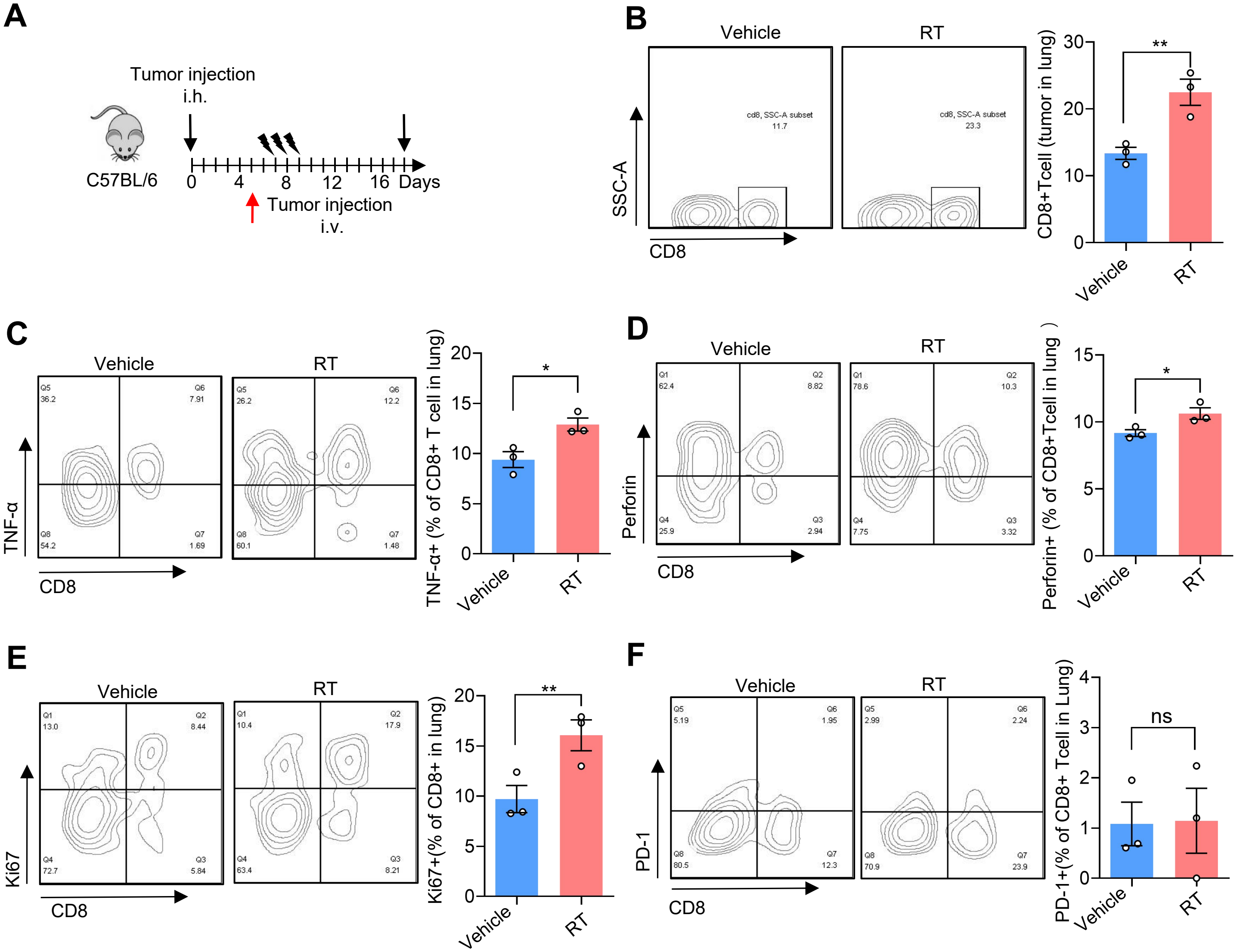

Figure S2 Effect of radiotherapy on CD8⁺ T cell infiltration in distant lung metastases. (A) Experimental diagram. C57/BL6 mice were subcutaneously inoculated with 1×10^6 B16F10 cells, and on day 5, they received intravenous injection of 1×10^6 B16F10-luc cells. On day 7, bioluminescence imaging was performed, followed by RT to the subcutaneous tumors in the RT group (8 Gy × 3 fractions) for consecutive 3 days. On day 18, bioluminescence imaging was performed again. (B) FCM analysis of $CD8^+$ T cell infiltration in melanoma tissues in the vehicle and RT groups. (C-F) FCM analysis of TNF-α, Perforin, Ki-67, and PD-1 expression in $CD8^+$ T cells in subcutaneous tumors of the vehicle and RT groups. Data are presented as mean ± SEM, n = 3. *p < 0.05, **p < 0.01, ***p < 0.001, ****p<0.0001 by Student's t-test.

Figure S3

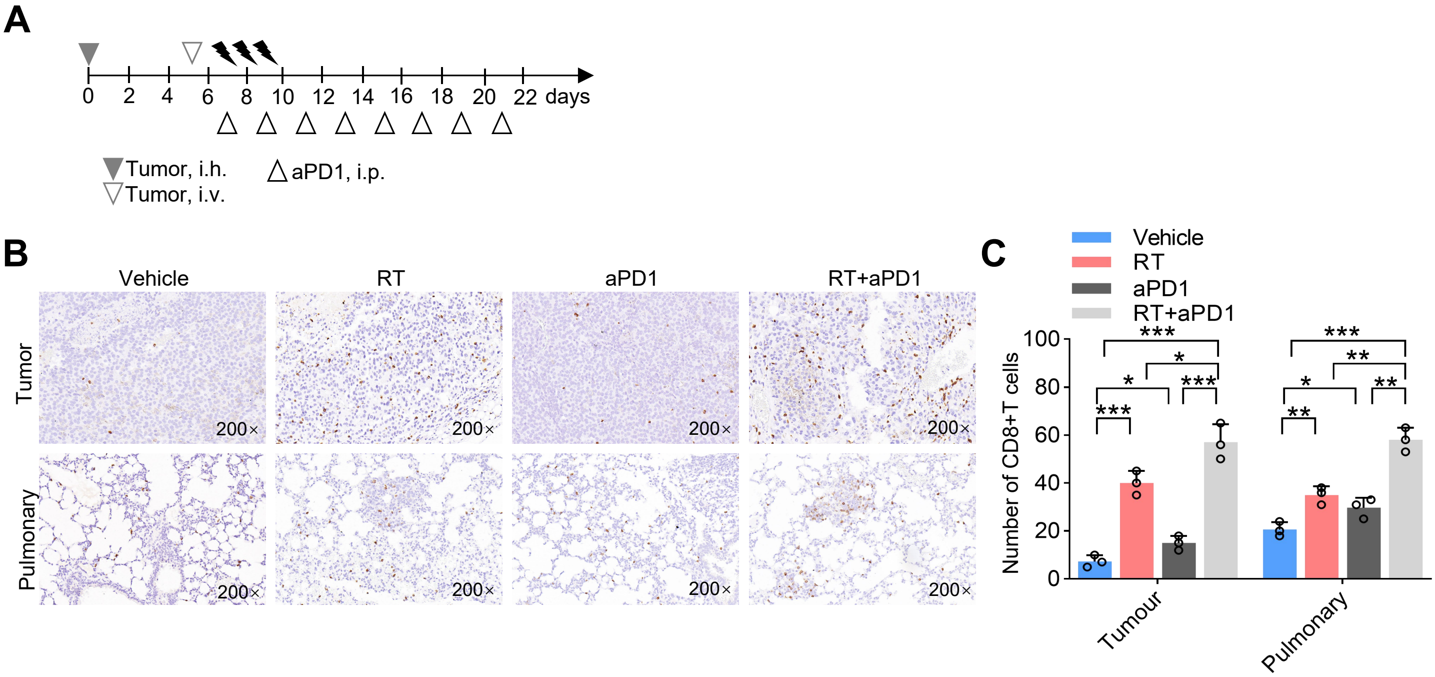

Figure S3 Infiltration of $CD8^+$ T cells in local tumors and distant lung metastases after combined radiotherapy and anti-PD1. (A) Experimental model of RT and anti-PD1 combination treatment. C57/BL6 mice were subcutaneously inoculated with 1×10^6 B16F10 cells. On day 5, 1×10^6 B16F10-luc cells were intravenously injected into the mice. RT was initiated on day 7 with a daily dose of 8 Gy for 3 consecutive days. Anti-PD1 was initiated on day 7 with intraperitoneal injections every 2 days at a dose of 100 µg per mouse until the observation endpoint. (B-C) Immunohistochemical analysis of $CD8^+$ T cell infiltration in subcutaneous tumors and lung metastases. Data are presented as mean ± SEM, n = 3. *$p < 0.05$, **$p < 0.01$, ***$p < 0.001$, ****$p<0.0001$ by two-tailed unpaired Student t-test.

Figure S4

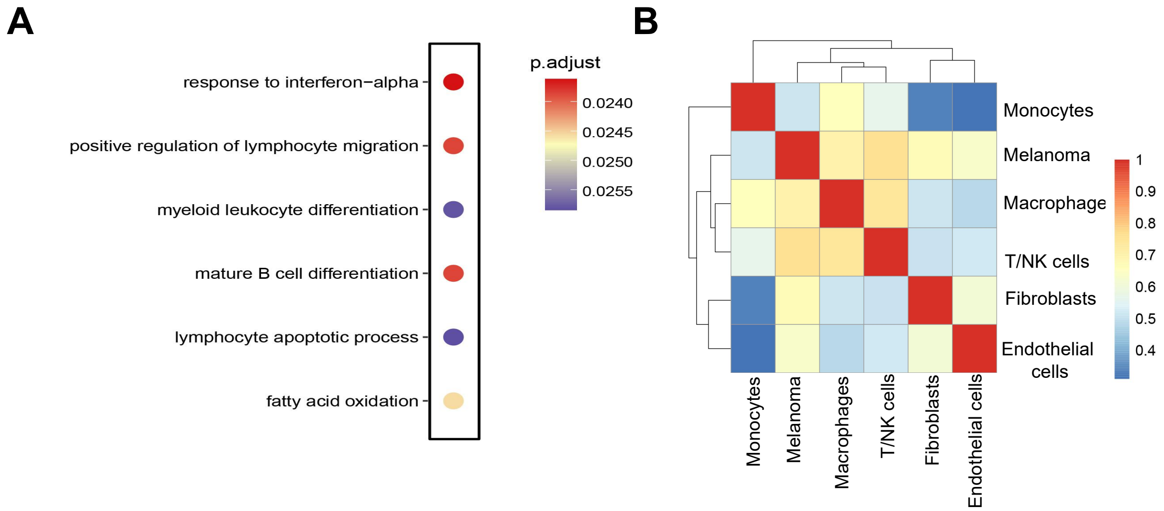

Figure S4 (A) Gene Ontology (GO) and Kyoto Encyclopedia of Genes and Genomes (KEGG) analyses were performed on differentially expressed genes between M1-like and M2-like macrophages, and the results were visualized using a bubble plot. (B) Spearman correlation analysis was conducted to assess the similarity of gene expression profiles among different cell types.

Figure S5

A

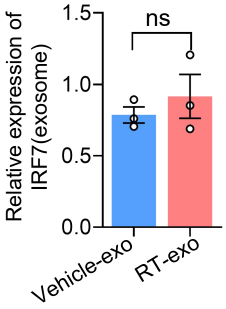

Figure S5 qRT-PCR was performed to measure the mRNA levels of IRF-7 in exosomes released by vehicle and RT-treated melanoma cells. Data are presented as mean ± SEM, n = 3. *p < 0.05, **p < 0.01, ***p < 0.001, ****p<0.0001 by Student's t-test.

Figure S6

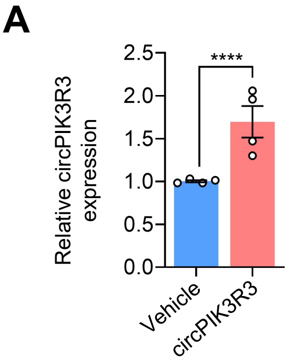

Figure S6 qRT-PCR was conducted to assess the overexpression efficiency of circPIK3R3 in Raw264.7 cells. Data are presented as mean ± SEM, n = 4. *$p < 0.05$, **$p < 0.01$, ***$p < 0.001$, ****$p<0.0001$ by Student's t-test.

Figure S7

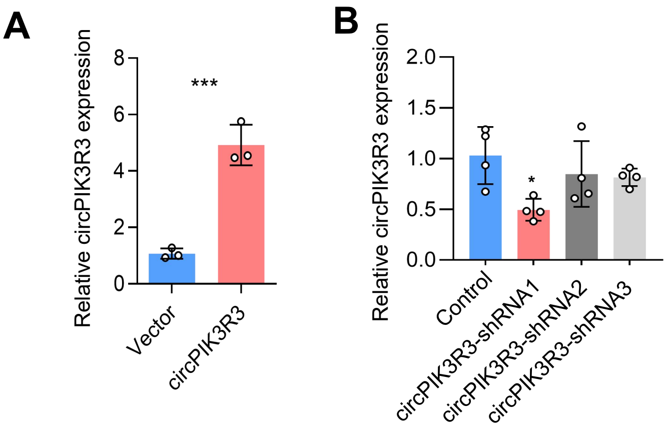

Figure S7 (A) qRT-PCR was used to evaluate the overexpression efficiency of circPIK3R3 in B16F10 cells. Data are presented as mean ± SEM, n = 3. *p < 0.05, **p < 0.01, ***p < 0.001, ****p<0.0001 by Student's t-test. **(B)** qRT-PCR was used to evaluate the knockdown efficiency of circPIK3R3 in B16F10 cells. Data are presented as mean ± SEM, n = 4. *p < 0.05, **p < 0.01, ***p < 0.001, ****p<0.0001 by two-tailed unpaired Student t-test.

Figure S8

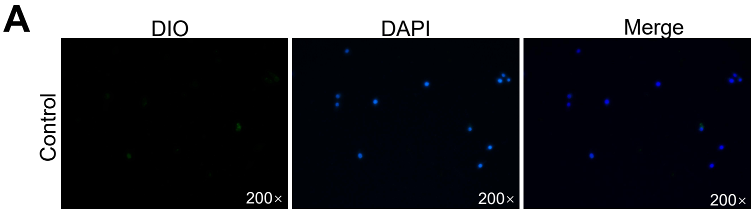

Figure S8 The control group without exosome treated with DiO. Raw264.7 were incubated with PBS-DiO (green) and counterstained with DAPI (blue).

Table S1 Sequences of Primers used for qRT-PCR.

IRF7	
Forward	5’-CACCACACTACACCATCTACCTG-3’
Reverse	5’-AGAGACTGTTGGTGCTAGACAAG-3’
**IL-1β**	
Forward	5’-CAGCACATCAACAAGAGCTTCAG-3’
Reverse	5’-GAGGATGGGCTCTTCTTCAAAGA-3’
**TNF-α**	
Forward	5’-GCCTCCCTCTCATCAGTTCTATG-3’
Reverse	5’-ACCTGGGAGTAGACAAGGTACAA-3’
**IFN-α**	
Forward	5’-TGTCTGATGCAGCAGGTGG-3’
Reverse	5’-AAGACAGGGCTCTCCAGAC-3’
**IFN-β**	
Forward	5’-GAGGAAAGATTGACGTGGGAGAT-3’
Reverse	5’-AGTCCGCCTCTGATGCTTAAAG-3’
**GAPDH**	
Forward	5’-ACTCTTCCACCTTCGATGCC-3’
Reverse	5’-TGGGATAGGGCCTCTCTTGC-3’
**mmu_circ_0000604**	
Forward	5’-TGGAGCTCCCTCTAAAATGTCTG-3’
Reverse	5’-TTACTCCCCAGGAAACTGCATC-3’
**mmu_circ_0011074**	
Forward	5’-GAGTACACAAGGACATCACAGCTCT-3’
Reverse	5’-TCATTTACCTCTTCCCTGGAAATAT-3’
**mmu_circ_0001249**	
Forward	5’-GATGAGTGGATGGCGAAGGA-3’
Reverse	5’-ATGGGGAACTCAATGATTTCATG-3’
**mmu_circ_0001415**	
Forward	5’-CTCCCAGTCCCTCCATAAACC-3’
Reverse	5’-TTCAGGCTCATCTTCTTCCCTT-3’
**mmu_circ_0013356**	
Forward	5’-GTAACAGCCAGTGTGCAGAAGAA-3’
Reverse	5’-TCATTTCAAACTTCTTAGCCAACA-3’
**mmu_circ_0000180**	
Forward	5’-CGCCTTGTTGAAGTGGTGTC-3’
Reverse	5’-TGGCACTTTCCATAGCAGGG-3’
**mmu_circ_0001894**	
Forward	5’-CACGGGAAGCTGGTCATCC-3’
Reverse	5’-GGTAGCTCCCGGACATGG-3’

Table S2 List of Primary Antibodies Used in the Study.

Antibody	Applications	Company
CD4	IF	CST (25229)
CD8	IF	Abcam (ab20975)
CD161	IF	CST(39197)
CD68	IF	santa cruz(sc-20060)
APC-CY7-CD45	F	BD Pharmingen(557659)
FITC -CD3	F	Biolegend(100203)
PerCP-Cy5.5-CD8	F	BD Pharmingen(551162)
BV421-CD279 (PD-1)	F	BD Pharmingen(562584)
APC-Ki67	F	BD Pharmingen(561126)
BV421-TNF-α	F	Biolegend(506327)
APC-Perforin	F	Biolegend(154403)
PerCP-Cy5.5-CD80	F	BD Pharmingen(560526)
APC-CD86	F	BD Pharmingen(558703)
PE-GZMB	F	eBioscience(2213195)
PE-CY7-IFN-γ	F	BD Pharmingen(557649)
P-STAT1	WB, IHC	Abcam(ab109461)
STAT1	WB	Abcam(ab239360)
p-JAK1	WB	CST (3331)
JAK1	WB	Abcam(ab133666)
IRF7	WB, IF	santa cruz(sc-74471)
GAPDH	WB	CST (5174)
GZMB	IHC	Abcam(ab255598)

Abbreviations: WB, western blot; IHC, immunohistochemistry; IF, immunofluorescence; F, flow cytometric analysis.

Table S3. Target sequences of circPIK3R3 shRNAs.

**shRNA**	**Target sequence**
CircPIK3R3	
shRNA-1	TCACAGCTCTTCCACCAAA
shRNA-2	AAGGACATCACAGCTCTTC
shRNA-3	ATCACAGCTCTTCCACCAA
